# Phenibut Addiction in a Patient with Substance Use Disorder

**DOI:** 10.7759/cureus.5230

**Published:** 2019-07-24

**Authors:** Kan Hong Zheng, Afra Khan, Eduardo D Espiridion

**Affiliations:** 1 Miscellaneous, West Virginia School of Osteopathic Medicine, Lewisburg, USA; 2 Psychiatry, Frederick Memorial Hospital, Frederick, USA

**Keywords:** phenibut, beta phenyl gamma aminobutyric acid, gaba, nootropic drug, alcoholism, alcohol withdrawal, substance use

## Abstract

Phenibut, a γ-aminobutyric acid (GABA) analog, is a synthetic, nootropic GABA_B_ receptor agonist used to treat anxiety, insomnia, alcohol withdrawal, and other conditions. The drug is licensed and widely used in Russia however, phenibut can be purchased through online vendors in other countries. The current literature on the effects of phenibut intoxication and withdrawal in humans is limited. In this case report, a 23-year-old male with a history of heavy phenibut and alcohol use presented to the emergency department with suicidal thoughts, somatic complaints, and seeking help with detoxification. His history and physical revealed symptoms indicative of alcohol withdrawal, but the extended period of his symptoms suggested an additive effect of his phenibut use. This unique case report illustrates how concurrent and heavy use of phenibut with alcohol may contribute to an extended and exacerbated withdrawal syndrome.

## Introduction

Nootropic drugs that can enhance cognitive function are becoming increasingly popular and available for purchase on the Internet. Phenibut, 4-amino-3-phenyl-butyric acid, is an analog of the γ-aminobutyric acid (GABA) neurotransmitter with an additional phenyl group that enhances diffusion across the blood-brain-barrier [1]. It was synthesized in the 1960s in Russia where it is licensed for use in anxiety, depression, attention deficit disorder, post-traumatic stress disorder, and other health issues. Marketed forms are currently synthesized as HCl, chloride (Baclofen), and citrate (Citocard) derivatives. Although the drug is not licensed in the United States, it is available through online vendors often marketed as a dietary supplement and cognitive enhancer [2]. However, research on the prevalence and effects of phenibut use is currently limited.

Phenibut taken at low doses (<20 mg/kg) is associated with nootropic effects and has no impairment in motor function. In contrast, high doses (>50 mg/kg) have tranquilizing effects and suppress motor activity, pain response, and induce altered electroencephalogram activity. Taken chronically, high doses promoted tolerance to the sedative effects over time. Chronic use has also been associated with psychosis and physical dependence [3, 2, 4].

The combination of phenibut with other pharmacological agents including antiparkinsonian agents, serotonin, and propranolol has been shown to have cooperative effects on the physiological outcome [5-7]. Thus, phenibut may have overlapping mechanisms of action with many pharmacological agents. Studies have suggested that phenibut may also have a protective role against alcohol-induced behavioral changes in animals and sleep disturbances in humans [8, 9]. Among its licensed uses in Russia, phenibut is used in the management of alcohol withdrawal [1]. However, we know little regarding how long-term phenibut use interacts with the effects of alcohol and regulates alcoholic behavior.

Alcohol use results in impaired central nervous system activity including poor motor coordination and behavioral changes due to altered synthesis, release, and signaling of neurotransmitters including serotonin, glutamate, GABA, and endocannabinoids as well as their corresponding receptors. Furthermore, the symptoms of phenibut withdrawal are similar to those of alcohol withdrawal, which include anxiety, agitation, depression, cognitive deficits, insomnia, hallucinations, and tremors [4, 10-13]. It is unknown whether the use and withdrawal symptoms associated with long-term phenibut use enhance those symptoms associated with concurrent alcohol use.

Here we present a case study of a heavy and long-term phenibut user who presented with increased withdrawal symptoms not typical of those observed in alcohol use.

## Case presentation

A 23-year-old Caucasian male with a history of illicit substance use self-presented to the emergency department (ED) of a community hospital with somatic complaints of feeling “the cells of his brain disappearing” and abdominal pain due to “feeling his gall bladder disappear.” He had visited the ED three additional times the same month for various complaints. He reported suicidal ideations with plans to overdose during this ED visit. While in the ED, the patient reportedly drank hand sanitizer. Nevertheless, the patient sought assistance with detoxification from alcohol and substance use. His reported alcohol consumption recently increased to include drinks one to five beers every day. He also began using phenibut, purchased online, in the past month. He stated that he would consume four to five grams per day. Upon attempting to stop consumption of these substances on his own, he reported experiencing symptoms of withdrawal, such as cold sweats, anxiety, difficulty sleeping, and visual and auditory hallucinations.

In addition, the patient reported recent symptoms of depression (sad mood, low energy level, anhedonia, feelings of worthlessness and helplessness), poor sleep, poor concentration, poor appetite, and weight loss. He admitted currently experiencing anxiety with ruminative worry, restlessness, racing thoughts, and panic attacks. His history is significant for recurrent suicidal thoughts since age 10 and a suicide attempt in which he stabbed himself several times in the neck with scissors while intoxicated during the previous year of this ED visit where he was hospitalized for a week following the episode. His history is also positive for a hospitalization and admissions to mental health treatment programs. His social history indicated a history of physical abuse by his stepfather at a young age and aggressive behavior.

The history was negative for psychotic and manic symptoms although a long pattern of illicit drug use remains a confounding factor in this interpretation. The patient admitted to experimentation with numerous drugs since age 13 including alcohol, cocaine, lysergic acid diethylamide (LSD), dimethyltryptamine (DMT), mushrooms (psilocybin), ketamine, ecstasy, opioids, inhalants, benzodiazepines, synthetic marijuana, amphetamines, and crystalline methamphetamine. In one instance, he had reportedly set himself on fire while under the influence.

Upon admission to the ED, the patient’s vitals and laboratory results were obtained. His vital signs, complete blood count, and basic metabolic panel were all within normal limits. The urine drug screen showed straw and clear-colored urine and was positive for cannabis. His salicylate level was less than 0.3 mg/dL, acetaminophen level was less than 5 mg/dL, and blood alcohol level was less than 10 mg/dL.

**Figure 1:**
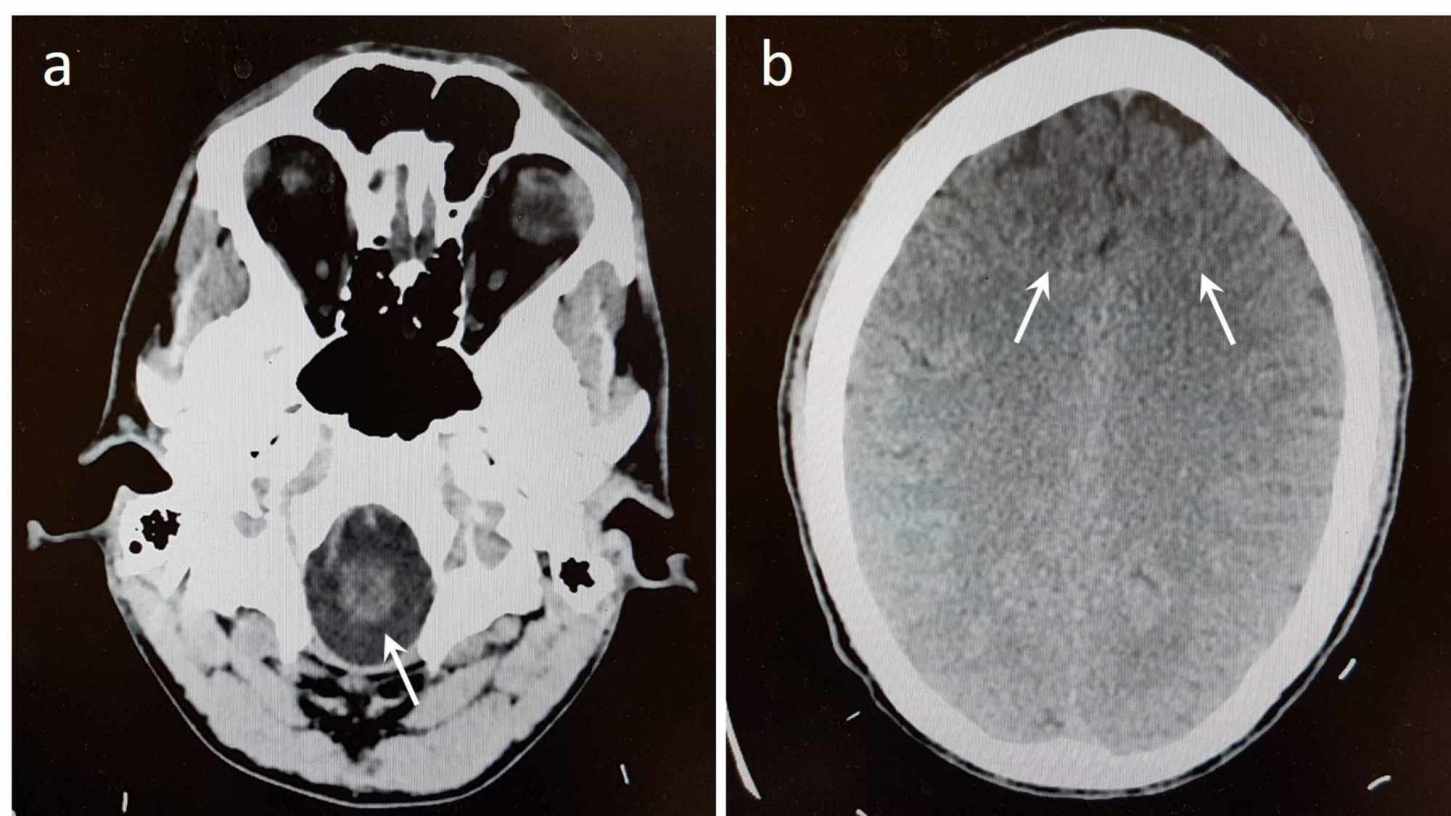
Magnetic resonance imaging (MRI) of the brain without contrast. MRI of the brainstem (a) and cortical regions (b) shows no lesions or structural abnormalities that would influence addictive behavior (arrows).

The results of administration of a mental status exam revealed cooperation during the interview; regular psychomotor activity; normal speech rate and volume; and logical, organized, and goal-directed thinking. He was alert and oriented to time, place, and person. The patient’s mood was reported to be depressed. His affect was anxious and labile, and new involuntary movements were noted. There were no suicidal or homicidal thoughts, plans, or intent at the time. He denied currently having auditory or visual hallucinations and reality testing was intact, but admitted to occasional auditory hallucinations where he hears ambulance sirens. There was no thought withdrawal or thought broadcasting. The patient’s intelligence was estimated as average and fund of knowledge appeared to be adequate. His insight and judgement were deemed to be limited with poor impulse control. His language was fluent. The patient was able to move all limbs with normal muscle tone and strength. MRI revealed no structural abnormalities that would result in abnormal behavior (Figure 1).

The patient was admitted to the Behavioral Health Unit of the hospital and the Clinical Institute Withdrawal Assessment protocol was utilized. Benzodiazepines were given for withdrawal symptoms of alcohol and phenibut. The patient was given Wellbutrin, 150 mg, by mouth daily for his depression symptoms and continued on Seroquel, 50 mg, at bedtime for further mood stabilization. Further, he was started on gabapentin, 100 mg, by mouth three times a day for impulsivity, anxiety, and protracted withdrawal symptoms.

## Discussion

In this report, we describe the case of a young male who experienced exaggerated psychiatric symptoms of alcohol withdrawal concurrent with phenibut use, a nootropic GABA derivative and neuromodulator. Research is currently limited regarding phenibut and its derivatives. This report adds to the growing number of clinical observations of the potentially harmful effects of unmoderated phenibut use in the general population.

Concurrent phenibut dependence with alcohol use

Despite the reported desired effects of reduced social anxiety and sense of euphoria, phenibut use has been associated with several adverse outcomes. The first reported case occurred in the United states in 2010 [10]. Since then the frequency of reported phenibut use has risen. Tolerance to the drug has been reported in as short as one to two weeks contributing to its potential addiction liability [14].

Several case reports have documented concurrent phenibut dependence and long-term alcohol use. A case report of a man with history of alcohol, opioid, and benzodiazepine use was reported consuming 8 g of phenibut per day for 10 months to self-medicate his anxiety and alcohol cravings. He was successfully treated with a 12-week baclofen taper [4]. Similarly, a man with a history of heroin use reported sedative-like intoxication from phenibut with a reported use of 100 g every week for four months [15]. Another case reported a male with a history of anxiety presenting at an emergency department for severe agitation and psychosis with reported phenibut addiction characterized with consumption of approximately 5 g daily for two months. His toxicology results were also positive for amphetamines [3]. Another patient began using low dose phenibut (0.1-0.3 g every few days) before escalating the dose in conjunction with heavy use of alcohol one week prior to admission to the emergency department. Upon ceasing use after arrival, the patient reported visual hallucinations and anxiety. The patient was successfully treated with a baclofen taper [12]. In all four cases, each patient had histories of prior or current drug use, including alcohol, as was observed in our patient.

Common biological pathways between phenibut and alcohol

While it as yet cannot be determined whether alcohol use exacerbates phenibut use or vice versa, there are common physiological mechanisms between the effects of phenibut and alcohol that could explain the common concomitant use of these two substances. Similar to alcohol, animal models have shown that a single phenibut dose stimulates dopamine production and the prevalence of its metabolites [16]. The increase in dopamine production may contribute to the euphoric experience obtained from both phenibut and alcohol.

Phenibut is a structural analog GABA, and an agonist of the GABA_B_ receptor. GABA receptors are inhibitory receptors that decrease neuronal excitability and probability of firing. Phenibut is also structurally related to baclofen, another GABA_B_ receptor agonist, which has been used to treat alcohol dependence as well as several cases of phenibut dependence [4, 11-13, 17, 18]. The opposing physiological outcomes of these two GABA_B_ agonists suggest either differences in pharmacological properties of the drug on the receptor or different off-target effects between the two compounds.

R-phenibut has also been shown to bind α_2_-δ voltage-gated Ca^2+^ channel subunit with four-fold greater affinity than to GABA_B_ receptors [19]. This was associated with anti-nociceptive properties similar to that of gabapentin, another GABA derivative which does not bind GABA_B_ receptors but reduces α_2_-δ channel currents. Thus, it has been presumed that phenibut binding also inhibits α_2_-δ channel currents. Gabapentin has been used successfully in the treatment of alcohol-use disorder [20]. Thus, the frequent concurrent use of phenibut with alcohol may be due in part to phenibut’s ability to reduce side effects associated with alcohol. Consistent with this, phenibut has been reported to reduce the effect of alcohol-induced behavioral and motor changes in an animal model [8].

## Conclusions

This case report demonstrates the importance of understanding how heavy phenibut use can impact one’s health, particularly when used concurrently with alcohol. It cannot be determined whether the phenibut use exaggerated the effect of the alcohol use or whether alcohol use exaggerated the effect of phenibut. Furthermore, the frequent use of phenibut and alcohol in combination may be to alleviate the adverse symptoms of phenibut use as tolerance develops from long term use. Our case report adds to the growing literature on the impact of phenibut and highlights the urgent need for more research on this compound.
